# Human U87 Astrocytoma Cell Invasion Induced by Interaction of βig-h3 with Integrin α5β1 Involves Calpain-2

**DOI:** 10.1371/journal.pone.0037297

**Published:** 2012-05-21

**Authors:** Jie Ma, Wei Cui, Shi-ming He, Yong-hong Duan, Li-jun Heng, Liang Wang, Guo-dong Gao

**Affiliations:** 1 Department of Neurosurgery, Tangdu Hospital, Forth Military Medical University, Xi'an, China; 2 Department of Endocrinology and Metabolism, Xijing Hospital, Fourth Military Medical University, Xi'an, China; 3 Department of Orthopaedic Surgery, 451 Hospital of Chinese People's Liberation Army, Xi'an, China; National Center for Scientific Research Demokritos, Greece

## Abstract

It is known that βig-h3 is involved in the invasive process of many types of tumors, but its mechanism in glioma cells has not been fully clarified. Using immunofluorescent double-staining and confocal imaging analysis, and co-immunoprecipitation assays, we found that βig-h3 co-localized with integrin α5β1 in U87 cells. We sought to elucidate the function of this interaction by performing cell invasion assays and gelatin zymography experiments. We found that siRNA knockdowns of βig-h3 and calpain-2 impaired cell invasion and MMP secretion. Moreover, βig-h3, integrins and calpain-2 are known to be regulated by Ca^2+^, and they are also involved in tumor cell invasion. Therefore, we further investigated if calpain-2 was relevant to βig-h3-integrin α5β1 interaction to affect U87 cell invasion. Our data showed that βig-h3 co-localized with integrin α5β1 to enhance the invasion of U87 cells, and that calpain-2, is involved in this process, acting as a downstream molecule.

## Introduction

Gliomas have a high incidence rate, and represent the most common form of primary intracranial tumors. They are generally malignant and highly invasive to surrounding structures, and prognosis is largely correlated with tumor stage. Because of these fatal characteristics, it is hard to perform complete resection by surgery. Although much work has been done to find clues as to invasive biomarkers and effective treatment methods, the molecular mechanisms need to be further investigated [1].

Transforming growth factor (TGF)-β-inducible gene-h3 (βig-h3) is widely expressed in various types of tumor cells. Though it is not normally expressed in tissues of the central nervous system, it was demonstrated to be expressed in U87 human astrocytoma cells [2], [3]. According to its molecular structure and functions, different names have been assigned to the protein, such as TGFBI, RGD-CAP, and MP78/70. Previous studies have demonstrated that by interacting with integrin α3β1 or regulating store-operated Ca^2+^ entry, βig-h3 promotes the migration and invasive ability of tumor cells [4], [5], [6]. However, the role of βig-h3 in affecting glioma cell invasion in the transduction pathway remains to be investigated.

Integrins are transmembrane heterodimers composed of α and β chains that provide physical and functional links between cell-cell and cell-ECM (extracellular matrix) interactions to mediate many cellular activities in tumors [7], [8], [9]. As we know, the interaction of integrins with ECM is related to cell viability and invasion. Proteins such as EMMPRIN (extracellular matrix metalloproteinase inducer) can interact with integrins to enhance the progression of hepatoma cells [10], [11]. In the present study, we found that βig-h3 co-localized with integrin α5β1 in U87 cells. However, very little information is available regarding the potential roles of this phenomenon. Given that βig-h3 and integrin can be involved in tumor invasion, the interaction of βig-h3 with integrin α5β1 may also affect the invasion of U87 cells.

Cell invasion is a characteristic of most malignant tumors, and in glioma cells this process is often mediated by calpain-2, a calcium-dependent thiol proteinase, which consists of a catalytic subunit and a regulatory subunit [12], [13]. It can be activated by millimolar levels of Ca^2+^ to enhance tumor invasion [14], [15], [16]. We presume that Ca^2+^ is the “key point” among βig-h3, integrin α5β1 and calpain-2, and therefore attempted to elucidate this relationship.

In the present study, we showed that βig-h3 and integrin α5β1 form a complex, and that they activate MMP secretion and enhance invasive potential via its downstream molecule calpain-2 in U87 cells.

## Results

### siRNA knockdowns can inhibit the expression of βig-h3 and calpain-2 in U87 cells

**Figure 1:**
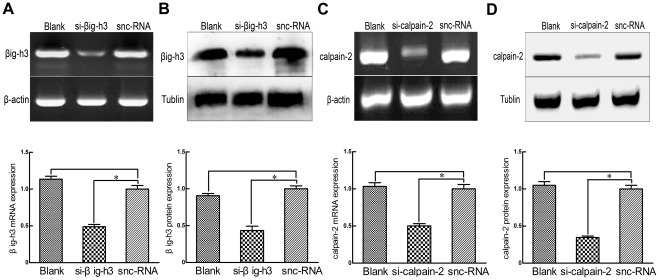
Effects of silencing βig-h3 and calpain-2 in U87 cells. Thirty-six hours after si-βig-h3 or snc-RNA transfection of U87 cells, RT-PCR (A) and western blotting (B) were performed to test the mRNA and protein levels of βig-h3. U87 cells treated with si-βig-h3 significantly reduced the mRNA expression of βig-h3 to 47.9%±4.1%, and reduced the protein expression of βig-h3 to 43.4%±6.5% in comparison to the snc-RNA treated cells (*P*<0.01). Thirty-six hours after si-calpain-2 or snc-RNA transfection of U87 cells, RT-PCR (C) and western blotting (D) were performed to test the mRNA and protein levels of calpain-2. U87 cells treated with si-calpain-2 significantly reduced the mRNA expression of calpain-2 to 51.1%±3.5%, and reduced the protein expression of calpain-2 to 34.6%±2.0% in comparison to the snc-RNA treated cells (*P*<0.01). Data are representative of three independent experiments. Bars represent the mean of triplicate samples and error bars represent standard deviation. *p<0.01 versus corresponding cells with snc-RNA treatment.

Previous studies have shown that βig-h3 and calpain-2 are expressed in U87 cells [2], [3], [12]. To obtain further information about their roles, small interfering RNAs (siRNAs) were transfected into U87 cells for 36 hours to knockdown βig-h3 and calpain-2 RNA and protein expression. Silencer negative control siRNAs (Snc-RNAs) were also used as a negative control, according to the manufacturer's protocol. As compared with snc-RNA treated cells, the siRNA knockdowns could effectively decrease the mRNA expression of βig-h3 and calpain-2 (47.9%±4.1% and 51.1%±3.5%, respectively), and the protein expression of βig-h3 and calpain-2 was significantly reduced to 43.4%±6.5% and 34.6%±2.0% (*P*<0.01, Figure 1A–1D).

### Downregulation of βig-h3 and calpain-2 decreases MMP secretion and U87 cell invasion

**Figure 2:**
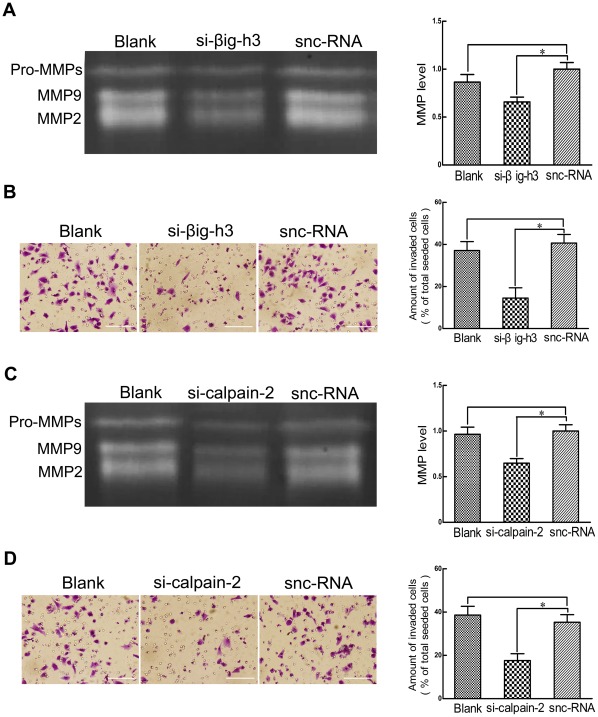
Effects of silencing βig-h3 and calpain-2 on MMPs secretion and invasion. Thirty-six hours after si-βig-h3 or snc-RNA transfection of U87 cells, Gelatin zymography (A) and invasion assay (B) were performed to test MMP secretion and invasive potential. Thirty-six hours after si-calpain-2 or snc-RNA transfection of U87 cells, Gelatin zymography (C) and invasion assay (D) were performed to test MMP secretion and invasive potential. Data are representative of three independent experiments. Bars represent the mean of triplicate samples and error bars represent standard deviation. *p<0.01 versus corresponding cells with snc-RNA treatment. Bar  = 100 um.

βig-h3 and calpain-2 promote invasion in tumor cells and they also enhance MMP secretion potential which is implicated in promoting metastasis by degradation of ECM. To further test for the ability to specifically suppress βig-h3 and calpain-2, we analyzed the expression level of MMPs and their invasive ability following knockdown of βig-h3 and calpain-2 in U87 cells. Snc-RNAs were also used as a negative control, according to the manufacturer's protocol. The results showed that these knockdowns reduced the density of MMPs and the invasive potential of U87 cells (*P*<0.01, Figure 2A–2D).

### βig-h3 co-localizes with integrin α5β1 on U87 cell membranes

**Figure 3:**
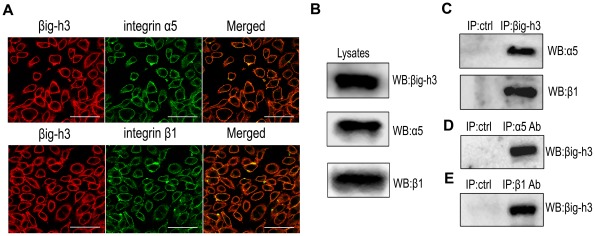
Expression and immunoprecipitation of βig-h3 and integrin α5β1 in U87 cells. (A) U87 cells were double-stained for βig-h3 (red) and integrin α5 or β1 (green). (B) Expression of βig-h3 and integrin α5 and β1 subunits in U87 cell lysates. (C) Precipitates from βig-h3 immunocomplexes were detected for precipitated integrin α5 and β1 subunits. Mouse IgG was used as a negative control. Precipitates from α5 (D) or β1 (E) immunocomplexes were detected for precipitated βig-h3. Mouse IgG was used as a negative control. Bar  = 50 um.

The invasive ability of human hepatoma cells is enhanced by the interaction of βig-h3 with integrin α3β1 [5]. According to the study, we hypothesized that βig-h3 might interact with integrin α5β1 to affect the invasive ability of U87 cells. So we performed immunofluorescent double-staining and confocal imaging analysis to examine cellular distribution. The results showed that staining overlapped diffusely throughout the surface of U87 cells (Figure 3A). To further confirm this result, Co-immunoprecipitation experiments were performed to detect the immunoreactivity of βig-h3 and the integrin α5 and β1 subunits. As Figure 3B–3E indicates, βig-h3 and integrin α5β1 interacted in their native conformations in U87 cell lysates.

### Interaction of βig-h3 with integrin α5β1 mediates MMP secretion and cell invasion potential

**Figure 4:**
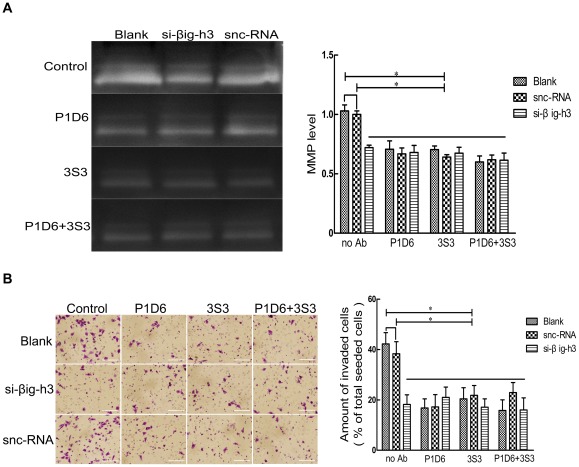
Invasive potential of U87 cells with or without integrin α5β1 mAbs and si-βig-h3 treatment. After treated with si-βig-h3 or snc-RNA, U87 cells were treated with P1D6, 3S3, or P1D6+3S3 for thirty-six hours. Gelatin zymography (A) and invasion assay (B) were performed to test MMP secretion and invasive potential. Data are representative of three independent experiments. Bars represent the mean of triplicate samples and error bars represent standard deviation. *p<0.01 versus corresponding cells with no antibody treatment. Bar  = 100 um.

To identify the function of the interaction of βig-h3 with integrin α5β1, antibodies against the integrin subunits and βig-h3 were used. Integrin α5β1, which is composed of an α5 chain and a β1 chain, is a receptor for fibronectin that is a component of ECM. It recognizes the sequence Arg-Gly-Asp (RGD) in its ligands. The “function blocking” antibodies, mouse anti-human α5 mAb (P1D6) and mouse anti-human β1 mAb (3S3), neutralize binding of α5β1 to the central cell adhesion domain of fibronectin. Such reaction may result in decrease of invasion potential and MMP secretion [17]–[20]. We treated U87 cells with P1D6, 3S3, P1D6+3S3 and si-βig-h3 for 36 h, and then examined the invasive ability and the presence of MMPs. As shown in Figure 4A and 4B, no significant cell number and MMP density changes were found between the snc-RNA transfected alone groups and the no antibody treated groups (*P*>0.01). As compared with the snc-RNA treated alone cells, invasive potential were markedly decreased after treated with P1D6, 3S3 or P1D6+3S3 (*P*<0.01), but no significant difference were found in the groups, including blank+P1D6, blank+3S3, blank+P1D6+3S3, snc-RNA+P1D6, snc-RNA+3S3 and snc-RNA+P1D6+3S3 groups (*P*>0.01). Similar results were observed in βig-h3 siRNA transfected alone groups compared with snc-RNA treated alone groups and the no antibody treated groups (*P*<0.01), but there were no significant differences in comparison with blank+P1D6, blank+3S3, blank+P1D6+3S3, snc-RNA+P1D6, snc-RNA+3S3 and snc-RNA+P1D6+3S3 groups (*P*>0.01). The addition of antibodies against α5 and/or β1 did not further reduce invasion or MMP secretion in βig-h3 siRNA transfected cells, compared with the cells treated with βig-h3 siRNA alone (*P*>0.01). These results indicated that βig-h3 and integrin α5β1 were both required for and dependent upon the invasion of U87 cells.

### Calpain-2 is a downstream signaling molecule of βig-h3-mediated invasion involving α5β1

**Figure 5:**
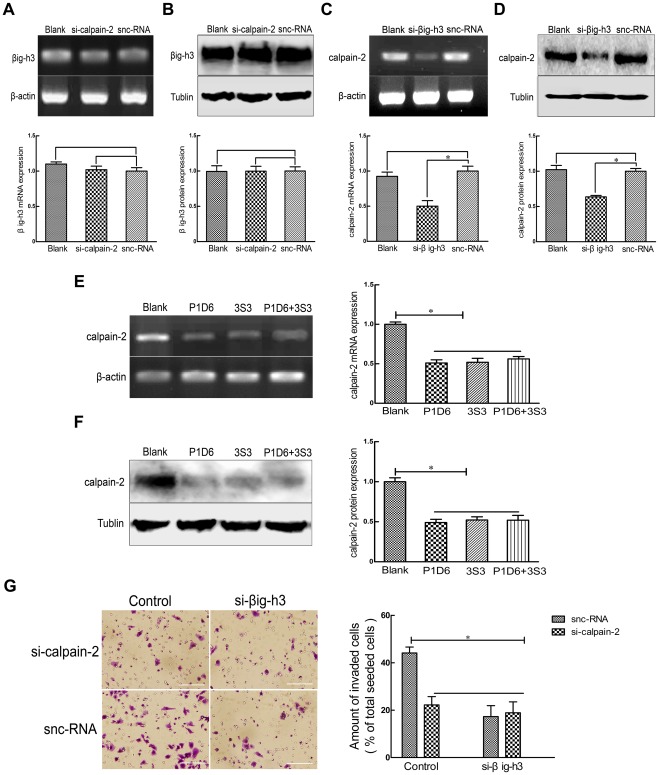
The expression of calpain-2 and βig-h3 after si-βig-h3, si-calpain-2 or integrin α5β1 mAbs treatment. Thirty-six hours after si-calpain-2 treatment, RT-PCR (A) and western blotting (B) were performed to test the mRNA and protein levels of βig-h3. Thirty-six hours after si-βig-h3 treatment, RT-PCR (C) and western blotting (D) were performed to test the mRNA and protein levels of calpain-2. Thirty-six hours after P1D6, 3S3 and P1D6+3S3 treatment, RT-PCR (E) and western blotting (F) were performed to test mRNA and protein levels of calpain-2. (G) Thirty-six hours after si-βig-h3 and si-calpain-2 treatment, alone or in combination, invasion assay was also performed to test invasive potential. Data are representative of three independent experiments. Bars represent the mean of triplicate samples and error bars represent standard deviation. *p<0.01 versus corresponding cells with snc-RNA or no antibody treatment. Bar  = 100 um.

We used western blotting analysis, Reverse transcriptase polymerase chain reaction (RT-PCR) assay and invasion assay to test if calpain-2 is upstream or downstream of βig-h3 and integrin α5β1. The results showed that the mRNA and protein expression of βig-h3 was not changed following calpain-2 siRNA knockdown (P>0.01, Figure 5A and 5B), but the mRNA and protein expression of calpain-2 was decreased when U87 cells were treated with βig-h3 siRNA, (P<0.01, Figure 5C and 5D). And the mRNA and protein expression of calpain-2 was decreased when U87 cells were treated with P1D6, 3S3 and P1D6+3S3 (P<0.01, Figure 5E and 5F). As compared with the snc-RNA treated alone groups, invasive potential was obviously decreased after treated with si-βig-h3, si-calpain-2 or si-βig-h3+si-calpain-2 (*P*<0.01), but no significant difference were found in the groups, including snc-RNA+si-βig-h3, si-calpain-2 and si-βig-h3+si-calpain-2 groups (*P*>0.01, Figure 5G). All of these results suggest that calpain-2 acts downstream of βig-h3 and α5β1 integrin to influence invasion of U87 cells.

## Discussion

Studies of the molecular mechanism of invasion could reveal targets for glioma treatment. βig-h3, a 68 kDa extracellular matrix protein mainly induced by transforming growth factor-β (TGF-β), was first identified in the human lung adenocarcinoma cell line A549 [21]. It is expressed in many cells and tissues including the heart, liver, stomach, skeletal muscle and kidney [6], [21], [22]. Although its roles are largely unknown, it has been suggested that it involves in the regulation of many aspects of tumor cell processes, including cell adhesion, spreading, invasion, proliferation and apoptosis [22]–[25]. It has also been associated with corneal dystrophy, wound healing, atherosclerosis and many other human diseases [2], [26]. It contains four repetitive FAS-1 domains and an integrin recognition site named RGD sequence which can serve as a ligand of integrins. In this study, we focused on the receptors for βig-h3, the roles of which still remain largely unknown and tested if they could influence the invasive ability of U87 cells. The results presented here show that βig-h3 enhances the invasive potential of U87 cells by interacting with integrin α5β1.

Integrins, a large family of cell matrix adhesion receptors, have been demonstrated to play important roles in many types of tumor cells. Through the interaction with the basement membrane, integrins can mediate adhesion and invasion [8], [25], [27]. Previous studies have shown that integrin α6β1 can enhance the aggressiveness of U87 glioma cells, and the apoptosis of Ntera2 neuronal cells, which are being evaluated to employ in central nervous system (CNS) transplantation, was delayed by the activation of integrin α5β1 [8], [28]. In particular, integrin α5-laminins can highly enhance the invasion of all types of glioma cells, and the migration of U251 glioma cells is downregulated by fibronectin, an ECM ligand of integrin α5β1 [29], [30]. Therefore, it was not surprising that integrin inhibitors serve as a potential drug to prevent tumor cell invasion. JSM6427 and SJ749, inhibitors of integrin α5β1, attenuate glioma cell proliferation and invasion [22], [31]. In the present study, we demonstrated that βig-h3 was positively related to the expression of integrin α5β1 and there are no previous reports about the signaling mechanism of βig-h3-integrin α5β1 interaction. We further observed that antibodies to α5 and/or β1 could effectively reduce βig-h3-mediated invasion and MMP secretion, and provided no significant additional inhibitory effect in U87 cells. Hence, we proposed a hypothesis that βig-h3 interaction with integrin α5β1 can regulate invasion of U87 cells.

βig-h3 plays key roles in tumor cell invasion and previous study has demonstrated that it increases Ca^2+^ influx to enhance secretion of MMPs [4]. Ca^2+^ is known to be involved in the motility, apoptosis, proliferation of cancer cells, as well as invasion [32], [33]. Focal adhesion kinase (FAK), mitogen-activated protein kinase (MAPK) and extracellular regulated protein kinase (ERK) are crucial components in the signaling pathways of some integrins, and all may influence Ca^2+^ accumulation. Specific inhibitors of these proteins can not only affect Ca^2+^ concentration and its signaling pathway, but significantly block integrin-induced or βig-h3-induced invasion and MMP release. Calpains are proteins that belong to the family of calcium-dependent intracellular cysteine proteases, and are ubiquitously expressed in glioma cells. These include μ- and m-isozymes, and are involved in the degradation of the main components of matrix and glycan, which are correlated with many diseases such as Alzheimer's and stroke [13], [34]. Downregulation of calpains after transfection with calpain-1 (μ-calpain) or calpain-2 (m-calpain) siRNA could reduce the secretion of MMPs and attenuate the adhesive and invasive potentials of some tumor cells [35], [36]. ERK and MAPK are upstream molecules of calpains, and FAK is a common substrate [37], [38]. Thus, Ca^2+^ might be a “medium” to affect the invasive ability promoted by βig-h3, integrin α5β1 and calpain-2, and the precise mechanism remains to be elucidated. We speculate that βig-h3 enhances the invasion potential of U87 cells via integrin α5β1-calpain-2 signaling pathways.

Our data demonstrates that βig-h3 activates MMP secretion and promotes invasive potential by interacting with integrin α5β1 via its downstream molecule calpain-2 in U87 cells. These results are significant in that they suggest that mechanisms regulating βig-h3-α5β1 interactions, and their role towards calpain-2 signaling may constitute a novel anti-glioma drug target.

## Materials and Methods

### Cell culture

Human U87 astrocytoma cell line, which was purchased from the Type Culture Collection of the Chinese Academy of Sciences (Shanghai, China), were routinely grown in Dulbecco's Modified Eagle's Medium (DMEM) plus 10% fetal bovine serum (FBS) at 37°C in a humidified atmosphere of 5% CO_2_ incubators.

### Gene silencing of βig-h3 and calpain-2

U87 cells were transfected with βig-h3 siRNA (si-βig-h3) and calpain-2 siRNA (si-calpain-2) using Lipofectamine 2000 reagent (Invitrogen, USA) according to the manufacturer's protocol. snc-RNA (Ambion, USA) was used as negative control under similar conditions. The sequences of siRNA duplex targeting βig-h3 were as following: 5′-CCUUUACGAGACCCUGGGATT-3′ and 5′-UCCCAGGGUCUCGUAAAGGTT-3′ (Ambion, USA). And the siRNA sequence for calpain-2 was: GCGAGGACATGCACACCAT (GenePharma, China). Silencing effects were examined by RT-PCR and western blotting analysis.

### RT-PCR

Thirty-six hours after siRNAs transfection, U87 cells were collected to verify the mRNA expression by RT-PCR. Total RNA was extracted using Trizol reagent (Invitrogen, USA) and first-strand complementary DNA (cDNA) was reverse transcribed using the ReverTra Ace kit (Toyobo, China) according to the guidelines. The cDNA was used as the template and was amplified by PCR using a specific primer set for βig-h3 and calpain-2, and β-actin was used as the internal control to normalize variances. All primers and probes were Synthesized by Shanghai Sangon Co. The primers used were as follows: βig-h3, 5′-CATTGAGAACAGCTGCATCG-3′ (forward) and 5′-AGTCTGCTCCGTTCTCTTGG-3′ (reverse); calpain-2, 5′-GCAGCCATTGCCTCCCTCAC-3′ (forward) and 5′-ACCTCCACCCACTCGCCGTA-3′ (reverse); β-actin, 5′-CCCAGCCATGTACGTTGCTA-3′ (forward) and 5′-TCACCGGAGTCCATCACGAT-3′ (reverse). PCR was carried out under the following conditions: 5min denaturation at 94°C, renaturation for 30 cycles at 94°C for 30s, 57°C for 30s, 72°C for 30s and 7 min extension at 72°C. The products were finally separated on 1% agarose gels and were analyzed by ultraviolet light.

### Western blotting analysis

Thirty-six hours after siRNAs transfection, the conditioned medium of the U87 cells was collected. After BCA protein assay, equal amounts of protein were separated on 10% sodium dodecyl sulfate polyacrylamide gel electrophoresis (SDS-PAGE). Proteins were separated by electrophoresis and transferred onto a polyvinylidene fluoride (PVDF) membrane (Millipore). After being blocked with 5% non-fat milk and washed with TBS-T, the immunoblots were incubated with the designated antibodies. Signals were detected using the Western-Light chemiluminescent detection system (Applied Biosystems). And β-actin was chosen as the internal control.

### Co-immunoprecipitation and western blotting analysis

For immunoprecipitation, the interaction of βig-h3 with integrin α5β1 was detected by the ProFoundTM Mammalian Co-Immunoprecipitation Kit (Pierce, USA). U87 cells were collected and lysed by M-per reagent. Lysates were incubated with mouse anti-human βig-h3 mAb or mouse anti-human α5 mAb (P1D6) (Chemicon) and mouse anti-human β1 mAb (3S3) (Santa Cruz). And then bound proteins were collected onto a coupling gel to elute. After that, by western blotting, the elution was resolved by SDS-PAGE, and then blotted and probed with the designated antibodies to detect integrin α5β1 or βig-h3. HRP-conjugated rabbit anti-goat IgG (Amersham Pharmacia) was used as the negative control.

### Immunofluorescence and confocal microscopy

For the detection of the location of βig-h3 and integrin α5β1, after placed onto glass coverslips overnight, U87 cells were fixed in 4% paraformaldehyde in (Phosphate Buffered Saline) PBS and then were pre-incubated with 1% bovine serum albumin (BSA) in PBS for 1h. Coverslips were treated with the primary antibodies (Santa Cruz) at a 1∶200 dilution in PBS for 1h in a humid chamber. Primary antibody-treated cells were washed in PBS and then incubated with fluorescein isothiocyanate (FITC)–conjugated donkey anti-goat secondary antibodies (Santa Cruz) at a 1∶500 dilution in PBS for 1 h. Cell nuclei were stained with DAPI (Biotium, USA) for 10 min at 37°C. Finally, the cells were enveloped with glycerol and observed by FV1000 laser scanning confocal microscope (Olympus).

### Invasion assay

The assay was performed using 24-well Transwell units with a polycarbonate filter (8-μm pore size, Millipore) coated on the upper side with Matrigel at a concentration of 5 mg/ml to form a thin layer. Each lower chamber contained 600 ml of 0.5% FBS as the chemoattractant. After siRNAs transfection, U87 cells were harvested to put into the upper chamber and then incubated for 36 h at 37°C in a humid atmosphere containing 5% CO_2_. After incubation, non-migrating cells on the top of the chambers were completely removed by a cotton swab. Cells that invaded into the lower chambers were fixed in 95% methanol for 5 min and then determined using a colorimetric crystal violet assay.

### Gelatin zymography

Thirty-six hours after siRNAs transfection, the sample protein, which was extracted from the conditioned media and was mixed with sample buffer, was separated by 8% acrylamide gels containing 0.1% gelatin. The gels were washed in 2.5% Triton X-100 (Sigma, USA) solution for 15mins twice at room temperature with gentle agitation to remove SDS and were then soaked into reaction buffer (0.05 mol/l Tris-HCl pH 7.5, 0.2 mol/l NaCl, and 0.01 mol/l CaCl_2_) at 37°C overnight. The gels were stained with Coomassie brilliant blue for 6 h and were then destained for 0.5 h. The expression and activation of MMP were shown by negative staining.

### Statistical analysis

Statistical analysis was performed using SPSS 13.0 statistical software. The results were expressed as mean values ± SD. And one-way ANOVA was used to evaluate the statistical significance among the groups. The differences were considered significant when *P*<0.01.
